# Supplementary material for: Intracellular ATP Concentration and Implication for Cellular Evolution
Source: Biology (Basel). 2021 Nov 12;10(11):1166. doi: 10.3390/biology10111166 (PMC8615055; doi:10.3390/biology10111166)
Supplement: Supplementary file 1 [file biology-10-01166-s001.zip › biology-1392865-supplementary.pdf]

Supplementary Materials:

Table S1. Concentrations of ATP in cells, tissues, and organs.

ATP (mM)	Organism	Specimen	Extraction Method / Solvent	Assay type	mM conversion factor	Page, ATP concentration found	Ref.
	**Cardiac Muscle**						
4	Dog	Heart, Lagendorff perfused	PCA, 0.4 N	HPLC	0.21 (p to ww)	4, Fig. 2A	9
7.5	Mouse	Heart, perfused	*In vivo*	^{31}P NMR		472, Fig. 4B	10
4.7	Pig	Heart, 5 g biopsies	PCA, 7.1 %	NADH		H1980, Table 3	11
25.65*	Rat	Heart	Extract	HPLC	0.22 (p to ww)	5	12
5.71	Rat	Heart, perfused working	Intact	^{31}P NMR	0.22 (p to ww)	217, Table 5	13
11.4	Rat	Heart, perfused	Tris-EDTA	Luciferase	0.22 (p to ww)	132, Fig. 7A & B	14
24*	Rat	Heart	PCA, 1 %	Luciferase		8, Fig. 4	15
16	Rat	Myo-cardium	PCA, 5 %	NADH	0.176 (p to ww)	1004, Fig.4	16
4.7	Rat	Heart Lagendorff perfused	PCA, 7 %, cold	TPNH		482, Table 4	17
11.9	Rat	Heart	Homogeni-zation	Luciferase	0.16 (p to ww)	388, Fig. 3D	18
6.3	Rat	Heart	PCA	HPLC		H1612, Table 3	19
2.5	Rat	Heart, perfused	PCA	^{13}C NMR	0.313 (dw to ww)	799, Table 2	20
6.9	Rat	Heart, perfused	PCA, 1.6 M	Luciferase	0.313 (dw to ww)	H1753, Fig. 6	21
7.5	Rat	Heart, Langendorff perfused	PCA	HPLC	0.313 (dw to ww)	H93, Fig. 2, sham	22
4.2	Rat	Heart, Langendorff perfused	PCA	HPLC	0.212 (dw to ww)	160, Fig. 4	23
14.6	Rat	Heart	EDTA, 40 mM	^{31}P NMR, ext. ref.		789, Table 1	24
4.1	Rat	Heart, biopsy	PCA, 0.4 M	HPLC		41, Table 1	25
**Skeletal Muscle**							
4.47	Amphibia	Frog, *Rana temporaria*, gastroc-nemius muscle	PCA, 6 %	TPNH		28, Table 2	26
3.5	Amphibia	Frog, *Rana temporaria*, sartorii muscle	*Ex vivo*	^{31}P NMR		714	27
3	Amphibia	Frog, gastroc-nemius	*Ex vivo*	^{31}P NMR		2588, Table 1	4
5.9	Amphibia	Toad, gastroc-nemius	*Ex vivo*	^{31}P NMR		2588, Table 1	4
8.05	Aves	Chicken, *Gallus gallus*, pectoralis muscle	PCA, 6 %	TPNH		28, Table 2	26

6.2	Aves	Chicken pectoralis muscle	*Ex vivo*	^{31}P NMR		2588, Table 1	4
4.2	Aves	Chicken pectoralis muscle	*Ex vivo*	^{31}P NMR		2588, Table 1	4
7.04	Aves	Pigeon, *Columba livia*, pectoralis muscle	PCA, 6 %	TPNH		28, Table 2	26
6.51	Aves	Starling, *Sturnus vulgaris*, pectoralis muscle	PCA, 6 %	TPNH		28, Table 2	26
7.23	Aves	Pheasant, *Phasianus colchicas*, pectoralis muscle	PCA, 6 %	TPNH		28, Table 2	26
6.95	Crustacea	Lobster, *Homarus vulgaris*, abdominal muscle	PCA, 6 %	TPNH		28, Table 2	26
6	Human	Vastus lateralis muscle	PCA, 6 %	NADH	0.25 (dw to ww)	2246, Table 1	28
5.5	Human	Quadriceps femoris muscle	PCA, 0.5 M, + EDTA, 1 mM	NADH	0.23 (dw to ww)	118, Table VII	29
5.83	Insecta	Locust, *Schistocera gregaria*, flight muscle	PCA, 6 %	TPNH		28, Table 2	26
6.03	Insecta	Cockroach, *Periplaneta americana*, flight muscle	PCA, 6 %	TPNH		28, Table 2	26
7.15	Insecta	Water bug, *Lethocerus cordofanus*, flight muscle	PCA, 6 %	TPNH		28, Table 2	26
8.51	Insecta	Roseschafer, *Pahnoda ephippiata*, flight muscle	PCA, 6 %	TPNH		28, Table 2	26
5.66	Insecta	Dung beetle, *Heliocopris* sp., flight muscle	PCA, 6 %	TPNH		28, Table 2	26
5.35	Insecta	Honey bee, *Apis mellifera*, flight muscle	PCA, 6 %	TPNH		28, Table 2	26
7.72	Insecta	Blowfly, *Calliphora vicinia*, flight muscle	PCA, 6 %	TPNH		28, Table 2	26

0.76	Mollusca	Snail, *Helix pomatia*, foot muscle	PCA, 6 %	TPNH	28, Table 2	26
6.63	Mollusca	Scallop, *Pecten maxiums*, snap muscle	PCA, 6 %	TPNH	28, Table 2	26
2.02	Mollusca	Sea mussel, *Mytilus edulis*, posterior adductor muscle	PCA, 6 %	TPNH	28, Table 2	26
5.3	Mouse	Extensor digitorum longus muscle	Perfused intact muscle	^{31}P NMR	7524, Table 3	30
3.3	Mouse	Soleus muscle	Perfused intact muscle	^{31}P NMR	7524, Table 3	30
9.52	Mouse	Hind limb	In vivo	^{31}P NMR	3, Table 1	31
4.99	Mouse	Thigh	PCA, 6 %	TPNH	28, Table 2	26
5.14	Pisces	Dogfish, *Scylliorhin-us canicula*, white muscle	PCA, 6 %	TPNH	28, Table 2	26
0.62	Rat	Myoblastic H9c2 cells	PCA	Luciferase	554, Fig. 5	32
6.1	Rat	Diaphragm muscle	Perfused intact muscle	^{31}P NMR	7524, Table 3	30
6.7	Rat	Extensor digitorum longus muscle	Perfused intact muscle	^{31}P NMR	7524, Table 3	30
4.3	Rat	Extensor digitorum longus muscle	0.02 N HCl, followed by additional 112.5 μL 3.0 N PCA HCl, 0.02 M, + PCA, 3 N, 112.5 μL	HPLC	179, Table 2	33
6.63	Rat	Gastroc-nemius and plantaris muscles	PCA, alcoholic	Fluorometric enzymolysis	C33, Fig. 1	34
5	Rat	Gastrocnemius muscle	PCA, 10 %	TPNH	644, Table2	35
7.75	Rat	Plantaris muscle	PCA, alcoholic	TPNH	C34, Table 1	34
6.23	Rat	Thigh	PCA, 6 %	TPNH	28, Table 2	26
4.9	Rat	Thigh muscle *in situ*	PCA 10 %	TPNH	235, Table 4	36
4	Rat	Soleus muscle	Perfused intact muscle	^{31}P NMR	7524, Table 3	30
5.9	Rat	Tensor fascia latae	Perfused intact muscle	^{31}P NMR	7524, Table 3	30
7.5	Rat	Fast-twitch muscle	In vivo	Bio+NMR	C544, Methods	37

7.5	Rat	Fast-twitch muscle	In vivo	Bio+NMR		C544, Methods	37
7.5	Rat	Fast-twitch muscle	In vivo	Bio+NMR		C544, Methods	37
7.5	Rat	Fast-twitch muscle	In vivo	Bio+NMR		C544, Methods	37
7.5	Rat	Fast-twitch muscle	In vivo	Bio+NMR		C544, Methods	37
7.5	Rat	Fast-twitch muscle	In vivo	Bio+NMR		C544, Methods	37
8	Skeletal muscle	Skeletal muscle	Perfused intact muscle	^{31}P NMR		717	38
	**Brain**						
2.98	Guinea pig	Cerebral cortex slices	PCA, 6 %	TPNH		528, Table 3	39
5.9	Mouse	Brain cells	Dissociated	Luciferase	0.117 (p to ww)	206, Table 6A	7
2.4	Mouse	Brain	PCA, 6 %	TPNH		24, Fig. 1	40
16*	Mouse	Brain	PCA, 0.4 M	HPLC		325, top right text	41
1.23	Rat	Brain	PCA, 0.4 M	HPLC		325, top right text	41
2.7	Rat,adult	Brain, /wet wt	PCA, 3 M	HPLC		1346, Table 1	42
2.7	Rat, adult	Brain, /protein	PCA, 3 M	HPLC		1346, Table 1	42
2.5	Rat, neonate	Brain, /wet wt	PCA, 3 M	HPLC		1346, Table 1	42
2.6	Rat, neonate	Brain, /protein	PCA, 3 M	HPLC		1346, Table 1	42
	**Liver**						
2.2	Human	Liver	Extract	Luciferase	0.21 (p to ww)	2082, Fig. 1A	43
15.8*	Human	Liver slices	PCA	Luciferase		3 bottom, Table 1	44
9.1*	Human	Liver slices	TCA, 10%	Luciferase		1488, Table 2	45
6.4	Human	Liver slices	TCA, 10%	Luciferase		1488, Table 2	45
8.3	Human	Liver slices	TCA, 10%	Luciferase		1488, Table 2	45
2.74	Rat	Liver, *in situ*	PCA, 6 %	Gevers and Krebs		106, Table 1	46
2.3	Rat	Liver, *in vivo*	PCA, 0.6 N	HPLC	0.2 (p to ww)	868, Fig. 1	47
0.28	Rat	Liver, *in vivo*	PCA, 0.6 N	HPLC	0.2 (p to ww)	1045, Fig. 1	48
3.4	Rat	Liver, flash frozen	PCA, 0.7 N	Spectro-photometry		518, Table III	49
2.5	Rat	Liver, flash frozen	PCA, 0.7 N	HPLC		1288, Table 1	50
2.3	Rat	Liver, flash frozen	PCA, 0.7 N	HPLC		1288, Table 1	50
2.1	Rat	Liver, flash frozen	PCA, 0.7 N	HPLC		1289, Table 3	50
2.2	Rat	Liver, flash frozen	PCA, 0.7 N	HPLC		83, Table 1	51

4.2	Rat	Liver	PCA	HPLC		4215, Fig. 1B	52
2.6	Rat	Liver	PCA, 6 %	TPNH		550	53
2.2	Rat, male	Liver	PCA, 0.5 M	HPLC		1534, Table 1	54
2.4	Rat, female	Liver	PCA, 6 %	TPNH		212, Fig. 2	55
0.64	Rat	Liver hepato-cytes	Extract	Luciferase		326, graph	56
	**Retina**						
5.9	Rat	Retina	Biolumines-cence assay kit	Luciferase	0.117 (p to ww)	E551, Fig. 4D	57
6.8	Rat	Retina	PCA 6%	NADH	0.117 (p to ww)	5/15	58
2.4	Rat	Retina	PCA	Luciferase	0.25 (dw to ww)	65, Table 2	59
2.5	Rat	Retina	TCA, 12%	TPNH	0.25 (dw to ww)	673, Table 1	60
3.08	Frog, *Rana pipiens*	Retina	PCA	Fluorometry	0.25 (dw to ww)	1471, Table 1	61
	**Other Tissues**						
0.43	Human	Whole male blood	TCA, 10%	Column chroma-tography		1235, Table II	62
0.43	Human	Whole female blood	TCA, 10%	Column chroma-tography		1235, Table III	62
1.2	Human	Erythro-cytes	PCA	Column chroma-tography		430, Table 1	63
1.1	Human	Erythro-cytes	PCA, 6 %	HPLC		262, Table I	64
1.9	Human	Lympho-cytes	PCA, 0.4 M	Radionuclide, HPLC		2935, Table 5	65
2.4	Human	Neuturo-phils	TCA, 12%	NADH	0.16 (p to ww)	185, Fig. 6	66
2.1	Human	Pancreatic acinar cells	Cultured acinar cell lysate	Luciferase	0.16 (p to ww)	219, Fig. 5d′	67
2.5	Human	Lens	*Ex vivo*	^{31}P NMR		549, Table 1	68
1	Mouse	Erythrocytes	Extract	Luciferase		C598, Fig 3B	69
40*	Pig	Neuro-vascular endothelial cells	Tris-HCl, 100 mM, + EDTA, 4 mM, pH 7.75	Luciferase	0.177 (p to ww)	625, Fig. 6D	70
1.4	Rabbit	Erythrocytes	PCA, 6 %	HPLC		264, Table II	64
3	Rabbit	Lens	Ex vivo	^{31}P NMR		549, Table 1	70
3.2	Rabbit	Lens	Ex vivo	^{31}P NMR		706, Table II	1
3.6	Rabbit	Reticulo-cytes	PCA, 6 %	HPLC		264, Table II	64
51.8*	Rat	Epididymal fat pads	PCA, 6 %	TPNH		200, Table 6	71
1.71	Rat	Kidney	PCA, 6 %	Gevers and Krebs		109, Table 4	46
2	Rat, male Lewis	Lung	Tris, 0.1 M + EDTA, 2mM, pH 7.5	HPLC	0.301 (dw to ww)	303, Table 1	72
168*	*Caenor-habditis elegans*	Nematode	Boiling water	Luciferase	0.2 (p to ww)	9, Fig. 2C	73

2.7	*Rana pipiens*	Oocytes	HCl	Luciferase		13912	74
	**Microorganisms**						
	Bacteria						
0.42	*Anacystis nidulans*	Axenic culture	Tris, 50 mM, pH 8, sonicated	Luciferase	0.313 (dw to ww)	1208, Table 2	75
1.85	*Anacystis nidulans*	Kratz and Myers medium	TCA, 5%	^{32}P radio assay		75, Table 2	76
0.92	*Anacystis nidulans*	Axenic culture	PCA	Luciferase	0.313 (dw to ww)	150, Table 2	77
8.2	*Azotobacter vinelandii,* a nitrogen-fixing bacterium	Growing diazotrophically	PCA, 1.6 M	Luciferase	551 mol wt ATP	1364, Table 1	78
11.8*	*Azotobacter vinelandii*	Phosphate-sufficient culture	PCA, 1.6 M	Luciferase		1364, Table 1	79
1.2	*Chlorella kessleri*	*Axenic culture*	PCA	Luciferase	0.313 (dw to ww)	150, Table 2	77
1.5	*Escherichia. coli* strain JM109 (DE3)	Strain JM109(DE3)	*In vivo* in culture	Fluorescent		Fig.4	80
6.9	*Escherichia coli*	Axenic culture	PCA, 0.4 M	^{14}C radio assay	1 g/3.333 ml p	423, Fig. 1	81
1.8	*Escherichia coli,* strain K12	Strain K12, minimal medium	Acetic acid, 1 N	^{14}C radio assay	0.313 (dw to ww)	791, Fig. 1 caption	82
2.6	*Klebsiella pneumon-iae*	*Harrison & Pirt medium*	PCA, 6.2%	NADH	0.313 (dw to ww)	649, Table 1	83
0.37	*Nostoc sp.*	*Axenic culture*	Tris, 50 mM, pH 8, sonicated	Luciferase	0.313 (dw to ww)	1208, Table 2	75
5.3	*Plankton*	*Bacteria, North Sea*	Millipore	Luciferase		41, Fig. 2	84
0.42	*Rhodo-bacter sulfide-philus*	*Strain W4 (DSM 1374)*	PCA, 3 M	Luciferase	0.313 (p to ww)	245, Table 3 caption	85
0.64	*Scenedes-mus obtusius-culus*	Axenic culture	PCA	Luciferase	0.313 (dw to ww)	150, Table 2	77
	Archaea						
2.9	*Methanosarcina barkeri*	*Strain Fusaro (DSM 804)*	PCA, 3 M	Luciferase	0.313 (p to ww)	220, Fig. 6	86
1.7	*Sulfolobus acido-caldarius*	Archae-bacterial thermoacidop hile	PCA, 20%	Luciferase	0.313 (p to ww)	6110, Fig. 3A	87
9.1	*Zymomonas mobilis*	Log-phase growth	H_2SO_4, 0.6 N	Luciferase	551 mol wt ATP	22, Table 8	88
	Eukaryota						
3.7	*Saccharo-myces carls-bergensis*	fresh harvested	7.5% PCA	Firefly-lantern		855, Table 1	89
2.6	*Saccharo-myces cerevisiae*	In culture		Nanosensor		37583	90
2.3	*Saccharo-myces cerevisiae*	In culture		Nanosensor		102	91

2.1	*Saccharo-myces cerevisiae*	In culture		Nanosensor		738, Table 1	92
0.98	*Euglena gracilis*	*Axenic culture*	PCA	Luciferase	0.313 (dw to ww)	150, Table 2	77

*Outlier, not used in statistical comparison computations. Abbreviations used throughout this table: 13C NMR, carbon-13 nuclear magnetic resonance; 14C, the radio-nuclide incorporated for quantification; 31P NMR, phosphorus-31 nuclear magnetic resonance; 32P, the 32P radio nuclide counted following two-dimensional thin-layer chromatography; Bio+NMR, biochemical and NMR assays; EDTA, ethylenediaminetetraacetic acid; (dw to ww), dry weight to wet weight; Fluorescent, quantification of a fluorescent protein introduced by transformation of the JM109(DE3) strain; HPLC, high-performance liquid chromatography; ext. ref., methylenediphosphonic acid used for an external spectroscopic reference signal; Luciferase, refers to a variety of commercial luciferin-luciferase analytical kits for the measurement of the ATP concentration; Millipore, collected on Millipore filters; NADH, nicotine-adenine dinucleotides coupled enzymatically to ATP with the resultant reduced form, NADH, quantified spectrophotometrically; Nanosensor, a fluorescing intracellular aptamer-based ATP nanosensor for fluorescence quantification; (p to ww), protein to wet weight; PCA, perchloric acid; TCA, trichloroacetic acid; TPNH, triphosphopyridine nucleotide coupled enzymatically to ATP with the resultant reduced form (TPNH) quantified spectrophotometrically; Tris, tris(hydroxymethyl)aminomethane. All ATP concentrations were obtained from individual discrete samples.
